# The non-random clustering of non-synonymous substitutions and its relationship to evolutionary rate

**DOI:** 10.1186/1471-2164-12-415

**Published:** 2011-08-16

**Authors:** Lisa G McFerrin, Eric A Stone

**Affiliations:** 1 Graduate program in Bioinformatics, North Carolina State University, Raleigh, NC, USA 27695-7566; 2 Department of Genetics, North Carolina State University, Raleigh, NC, USA 27695-7614

## Abstract

**Background:**

Protein sequences are subject to a mosaic of constraint. Changes to functional domains and buried residues, for example, are more apt to disrupt protein structure and function than are changes to residues participating in loops or exposed to solvent. Regions of constraint on the tertiary structure of a protein often result in loose segmentation of its primary structure into stretches of slowly- and rapidly-evolving amino acids. This clustering can be exploited, and existing methods have done so by relying on local sequence conservation as a signature of selection to help identify functionally important regions within proteins. We invert this paradigm by leveraging the regional nature of protein structure and function to both illuminate and make use of genome-wide patterns of local sequence conservation.

**Results:**

Our hypothesis is that the regional nature of structural and functional constraints will assert a positive autocorrelation on the evolutionary rates of neighboring sites, which, in a pairwise comparison of orthologous proteins, will manifest itself as the clustering of non-synonymous changes across the amino acid sequence. We introduce a dispersion ratio statistic to test this and related hypotheses. Using genome-wide interspecific comparisons of orthologous protein pairs, we reveal a strong log-linear relationship between the degree of clustering and the intensity of constraint. We further demonstrate how this relationship varies with the evolutionary distance between the species being compared. We provide some evidence that proteins with a history of positive selection deviate from genome-wide trends.

**Conclusions:**

We find a significant association between the evolutionary rate of a protein and the degree to which non-synonymous changes cluster along its primary sequence. We show that clustering is a non-redundant predictor of evolutionary rate, and we speculate that conflicting signals of clustering and constraint may be indicative of a historical period of relaxed selection.

## Background

For functional biological sequences, and for proteins in particular, similarity in sequence is often predictive of similarity in structure and function. This has great utility, because while it is challenging to glean knowledge of structure and function, sequence information is comparatively easy to obtain. For this reason, and because comparing two sequences in an alignment is straightforward, pairwise alignments are often the first step toward annotating a sequence whose folded structure and biological function are unknown. When two sequences show extensive similarity and one of the two has been annotated, transferring that annotation provides an easy functional prediction; however, even in the complete absence of annotation, alignments can be used to ascribe functional importance to sites and regions in a sequence [1]. Consider, for example, two distantly-related sequences, say a pair of orthologous genes in human and chicken. Both the coding sequences of these genes and the amino acid sequences that they encode may be very different, yet particular stretches of residues may be well conserved [2]. While such surprisingly similarity can arise by random chance, it may also be the footprint of purifying selection, indicating a region of the sequence that is functionally important and resistant to evolutionary change.

**Figure 1:**
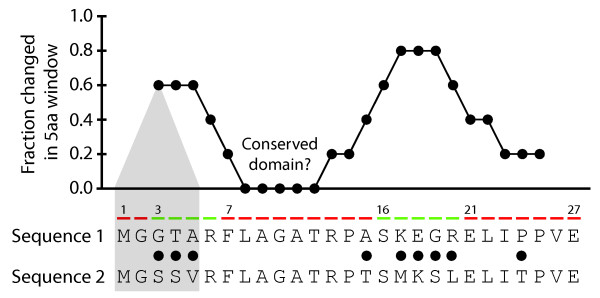
**Illustration of simple *de novo *annotation**. Shown is a comparison of two aligned protein orthologs, each of which is 27 amino acids in length. Filled circles between the sequences indicate sites at which the amino acids are distinct The sequence has been segmented into red (more slowly evolving) and green (more rapidly evolving) regions to illustrate the biological motivation. The figure above the alignment shows, for each of positions 3 through 25, the fraction of mismatched amino acids among positions *j *- 2 through *j *+ 2 plotted as a function of *j *(highlighted for *j *= 3 in gray). The region from positions 8 to 12 shows a deficit of changes, suggesting the possible presence of a conserved domain.

In proteins, functionally and structurally important residues are often organized into domains. Thus, in a comparison of related sequences, domains may be apparent as regions of surprising similarity. This style of *de novo *annotation is exploited routinely and underlies a number of web-accessible methods including but not limited to the Evolutionary Trace (ET) [3-5] and Evolution-Structure-Function analysis (ESF) [6,7]. The success of these methods relies upon two general characteristics of protein sequences, namely (1) that there exists heterogeneity among the rates at which sites in a protein evolve and (2) that the rates are spatially autocorrelated (see Figure 1). Consequently, more sophisticated *de novo *annotation schemes gain resolution through a combination of improved evolutionary models, accounting for site autocorrelation, and respecting spatial proximities induced by tertiary structure, e.g. [8-10].

Just as surprising regional similarity in a pairwise comparison may be of biological interest, interesting biology may be responsible for regions that are surprisingly distinct. For example, in a comparison of closely-related species, say human and chimpanzee, one expects a great deal of sequence similarity. In such a background, sometimes regions of surprisingly dissimilarity may encode positively selected adaptations, including those that have helped to distinguish us from our primate cousins [11]. Within a protein-coding gene, there is evidence that sites undergoing diversifying positive selection, that is those evolving more rapidly than the rate of neutral evolution would predict, cluster non-randomly along the primary sequence [12,13]. The web-accessible tool SWAKK, which is similar in spirit to ET and ESF, exploits this non-random distribution to identify positively-selected regions within a protein [14].

Synthesizing the above, there is evidence that both negative purifying selection and positive diversifying selection promote the clustering of amino acid differences in a pairwise comparison of protein sequences. By contrast, in the absence of selection at the protein level (e.g. for a pseudogene or fully redundant duplicate), clustering is not expected, unless for example the mutation process is biased or there is selection on the encoding DNA. In a snapshot of evolutionary time, most proteins are under purifying selection, whereby non-synonymous mutations that change the encoded protein are more likely to fix if they affect regions of the sequence of functionally lesser importance. This raises the possibility that for proteins under stronger purifying selection the clustering of amino acid differences in a pairwise comparison is more intense. To explore this and other possibilities, we introduce a simple statistic that quantifies the degree to which non-synonymous changes are clustered in a pairwise alignment.

In this manuscript, we consider aligned pairs of putatively orthologous protein-coding sequences across a variety of species. Within that focus, we hypothesize that: (1) there exists a genome-wide trend relating the intensity with which purifying selection acts on a protein sequence to the intensity with which non-synonymous changes are clustered in a pairwise alignment; (2) gene pairs which have undergone periods of relaxed or reversed constraint, such as might occur subsequent to gene duplication, appear as deviations from the genome-wide trend; and (3) the intensity with which non-synonymous changes are clustered in a pairwise alignment is a strong non-redundant predictor of evolutionary rate. Using our new "dispersion ratio" statistic, we provide evidence in support of each hypothesis as well as show that the hypotheses are robust to the choice of genomes compared.

## Methods

### Genome-wide pairwise comparisons of selection and dispersion

**Figure 2:**
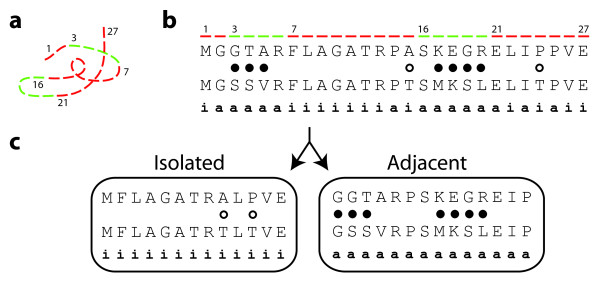
**Construction of the dispersion ratio**. (**a**) The protein sequence of 27 amino acids in length introduced in Figure 1. (**b**) The pairwise alignment introduced in Figure 1. Sites adjacent to sites at which the amino acids are distinct are labeled with an "**a**"; the remaining sites are labeled "**i**" for isolated. This time, filled circles denote amino acid differences at adjacent sites, whereas the circles indicating amino acid differences at isolated sites are hollow. (**c**) The alignment is partitioned into its isolated and adjacent constituents, and the selection parameter ω is estimated for each (as *ω_I _*and *ω_A_*, respectively). The dispersion ratio *ρ *is computed as *ω_I_*/*ω_A_*.

We obtained from Ensembl 46 pairwise codon alignments of all one-to-one orthologous protein coding sequences between human and eight other species: *Pan troglodytes, Macaca mulatta, Mus musculus, Rattus norvegicus, Canis familiaris, Monodelphis domestica, Gallus gallus, and Danio rerio*. As illustrated in Figure 2, we identified the sites in each alignment at which the encoded amino acids were distinct; these comprise the visible subset of all sites where a non-synonymous change has taken place. We labeled as "adjacent" (**a**) all sites adjacent to, but not necessarily including, any site identified as non-synonymous by amino acid comparison; the remaining sites were labeled "isolated" (**i**). The complete alignment was then partitioned into its adjacent and isolated components, yielding two disjoint subalignments. Within each genome-wide comparison, individual proteins were excluded from consideration unless the two subalignments each contained both a transition and a transversion event.

We used the method of Yang and Nielsen [15], as implemented in PAML (yn00, version 3.15), to estimate *Ka *and *Ks *for each complete alignment (no partitioning) and its two subalignments. The subalignment *Ka *and *Ks *estimates were denoted *Ka_A _*and *Ks_A_*, for the Adjacent alignment, and *Ka_I _*and *Ks_I_*, for the Isolated alignment. We obtained from PAML the standard errors for each estimate as well. We computed *ω *= *Ka*/*Ks *for the complete alignment, *ω_A _*= *Ka_A_*/*Ks_A _*for the adjacent alignment, and *ω_1 _*= *Ka_I_*/*Ks_I _*for the isolated alignment. The dispersion ratio was calculated as *ρ *= *ω_I_*/*ω_A_*. Within each genome-wide comparison, individual proteins were again excluded when either log(*ω*) or log(*ρ*) had a standard error greater than one. Standard errors for *ρ *and ω were approximated using the delta method as

$$SE_{\rho }=\sqrt{{\left(\frac{SE_{Ka_{I}}}{Ka_{I}}\right)}^{2}+{\left(\frac{SE_{Ks_{I}}}{Ks_{I}}\right)}^{2}+{\left(\frac{SE_{Ka_{A}}}{Ka_{A}}\right)}^{2}+{\left(\frac{SE_{Ks_{A}}}{Ks_{A}}\right)}^{2}}\;\text{and}\;SE_{\omega }=\sqrt{{\left(\frac{SE_{Ka}}{Ka}\right)}^{2}+{\left(\frac{SE_{Ks}}{Ks}\right)}^{2}}.$$

### *Saccharomyces *data and analysis

We obtained from Kellis et al. [16] the protein-coding genes and ortholog assignments (grouped by ORFs with unambiguous correspondence) for four *Saccharomyces *species: *S. cerevisiae, S. paradoxus, S. mikitae*, and *S. bayanus*. We considered only those proteins for which all four sequences were present, and these were aligned using ClustalW and subjected to phylogenetic analysis assuming the fixed unrooted topology ((*S. cerevisiae, S. paradoxus*), (*S. mikitae, S. bayanus*)). The method of Goldman and Yang [17], as implemented in PAML (codeml; version 3.15), was used to jointly infer "ancestral" sequences at the coalescence of *cerevisiae*/*paradoxus *and of *mikitae*/*bayanus*. This facilitated five pairwise comparisons that collectively span the tree: (1) *cerevisiae *vs. *cerevisiae*/*paradoxus*, (2) *paradoxus *vs. *cerevisiae*/*paradoxus *, (3) *mikitae *vs. *mikitae*/*bayanus*, (4) *bayanus *vs. *mikitae*/*bayanus*, and (5) *cerevisiae*/*paradoxus *vs. *mikitae*/*bayanus*. Subsequently, *Ka_A_, Ks_A_, Ka_I _*and *Ks_I _*were calculated for each. To compute a dispersion ratio for the tree, we first summed each of these measures across the five branches comprising ((*S. cerevisiae, S. paradoxus*), (*S. mikitae, S. bayanus*)). The dispersion ratio for each gene was thus given by (Σ*Ka_I_*/Σ*Ks_I_*)/(Σ*Ka_A_*/Σ*Ks_A_*) where each sum ranges over the five aforementioned pairwise comparisions.

### Comparing selection and dispersion for genes under recent positive selection

Within the human/chimpanzee dataset gathered from Ensembl, we identified those genes implicated as being under positive selection in the human lineage [18]. We then fit the model *Y_i _*= *α *+ *βX_i _*+ *γP_i _*+ *ε_i_*, where the response variable *Y_i _*is the log(*ω*) value for gene *i*, the continuous predictor variable *X_i _*is the log(*ρ*) value for gene *i*, and

$$P_{i}=\left\{\begin{array}{cc} 1, & if\;gene\;i\;was\;under\;recent\;positive\;selection\\ 0, & otherwise \end{array}\right)$$

### Comparing the dispersion ratio to established correlates of evolutionary rate

Measures of protein-related attributes in *Saccharomyces cerevisae *were collected from various sources (see Table 1). Careful attention was paid to ensure that we chose exclusion criteria and data transformations consistent with published studies. After exclusion and transformation, each of the protein-related attributes described above was investigated for correlation to both log(ω) and log(*ρ*) (Table 1, r_log(*ω*), X _and r_log(*ρ*), X_, respectively). Partial correlations were computed between log(ω) and log(*ρ*) after controlling for each of the protein-related attributes individually (Table 1, r_log(*ω*), log(*ρ*)|X_).

**Table 1:** Correlation and partial correlation between log(ω) and various protein attributes

Attribute (X)	N	**r**_**log(*ω*), X**_ (p-value)	**r**_**log(*ρ*), X**_ (p-value)	**r**_**log(*ω*), log(*ρ*)|X**_ (p-value)	Reference
**log(*ρ*)**	2,897	0.40077295 (0)	-	-	

**mRNA expression**	2,701	-0.3807253 (6.7e-94)	-0.2072764 (1.34e-27)	0.3558986 (0)	[20]

**Protein abundance**	1,930	-0.3878717 (2.572e-70)	-0.1586315 (2.4e-12)	0.3727785 (0)	[21]

**Codon adaptation index**^**1**^	2,895	-0.3741477 (7.23e-97)	-0.1898758 (6.63e-25)	0.3621437 (0)	[22]

**Codon adaptation index**^**2**^	2,643	-0.4055142 (3.568e-105)	-0.2027786 (6.31e-26)	0.3558753 (0)	[23]

**Dispensibility**^**1**^	1,562	0.1832102 (2.94e-13)	0.09173406 (0.000283)	0.3922312 (0)	[24]

**Dispensibility**^**2**^	49	-0.2296285 (0.1124)	0.01099192 (0.94025)	0.4143947 (0.00201)	[25]

**Sequence Length**	2,895	-0.01921694 (0.301313)	-0.01095773 (0.5556)	0.4006604 (0)	[26]

**Degree**	674	-0.1502817 (8.98e-5)	-0.0850535 (0.02724)	0.3938752 (0)	[27]

**Centrality**	674	-0.0193294 (0.616415)	-0.03150676 (0.414129)	0.4004375 (0)	[27]

**Contact density**	84	0.1411473 (0.2003)	0.05072567 (0.646781)	0.3981061 (9.39e-5)	[28]

**Fraction buried 25%**	84	0.2146396 (0.04992)	0.184923 (0.09218)	0.3761856 (0.000258)	[28]

**SS (helix)**	84	-0.1465735 (0.18337)	0.01745651 (0.8748)	0.4077974 (5.8299e-5)	[28]

**SS (strand)**	84	0.05027238 (0.64973)	-0.05152868 (0.6416)	0.4044114 (6.90152e-5)	[28]

**SS (turn)**	84	0.07785531 (0.48147)	-0.05314373 (0.6311)	0.406718 (6.1537e-5)	[28]

**SS (coil)**	84	-0.2148053 (0.04973)	-0.02548217 (0.818)	0.4048788 (6.743566e-5)	[28]

## Results

### The dispersion ratio as a simple measure of clustering

In this section we introduce the dispersion ratio, a measure of the degree to which non-synonymous changes are clustered in a pairwise alignment. The dispersion ratio thus quantifies spatial heterogeneity, which is in general a common and well-studied phenomenon. To illustrate how we have adapted the concept, in Figure 2 we present a hypothetical 27aa protein sequence that is composed of alternating rapidly- and slowly-evolving segments. To construct the dispersion ratio from a pair of aligned protein-coding sequences, we begin by identifying all positions *j *in the alignment at which the amino acids disagree. We then label the sites adjacent to mismatches (i.e. sites *j *- 1 and *j *+ 1 for each such *j*) with an "**a**". We next partition the alignment into two subalignments: one composed exclusively of the sites labeled "**a**", and one composed of the remaining sites, which we label "**i**" for isolated. Within each of these subalignments, we compute the ratio of the rate of non-synonymous substitutions to the rate of synonymous substitutions (*ω_I _*and *ω_A _*for the isolated and adjacent subalignments, respectively). The dispersion ratio *ρ *is the ratio of ratios *ω_I_*/*ω_A_*. The dispersion ratio measures the degree to which non-synonymous changes are clustered along a protein's primary sequence. It specifically quantifies the propensity for non-synonymous changes to neighbor one another in a comparison of homologous proteins. The philosophy of *ρ *can be conveyed through Figure 2 by simply tallying where the non-synonymous changes fall; there 2 of 13 isolated sites (15%) harbor a non-synonymous change, as compared to 7 of 14 adjacent ones (50%), suggesting a dispersion ratio smaller than one. As the name implies, larger values of *ρ *indicate that non-synonymous changes are more dispersed, whereas smaller values indicate a greater degree of clustering. Supplied with this definition of *ρ*, we can rephrase our first hypothesis as follows: if ω is the ratio of the rate of non-synonymous substitutions to the rate of synonymous substitutions for the entire protein, then we hypothesize a genome-wide trend that relates ω to *ρ*.

### A significant log-linear relationship between selection and dispersion

**Figure 3:**
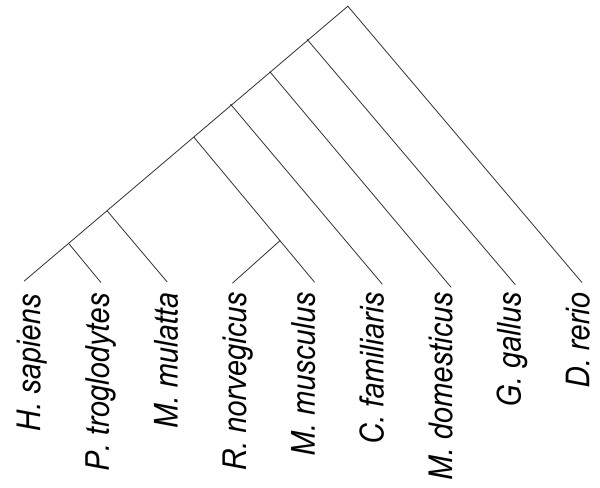
**Phylogeny of the eight species considered in pairwise comparisons**. From left: human, chimpanzee, macaque, rat, mouse, dog, opossum, chicken, and zebrafish.

**Figure 4:**
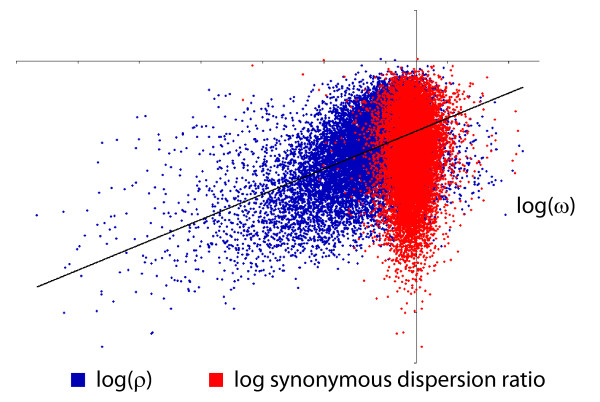
**Relationship between measures of selection and dispersion**. In blue, log(ω) is plotted against log(*ρ*) for aligned pairs of orthologous proteins shared between human and mouse (N = 11,894). The plot in red features the same *y*-axis but shows the synonymous dispersion ratio (see text) on the *x*-axis. The linear trend for log(*ρ*) vs. log(ω) is highly significant (black line; p < 2.2e-16) whereas the plot in red shows no significant linear trend.

**Figure 5:**
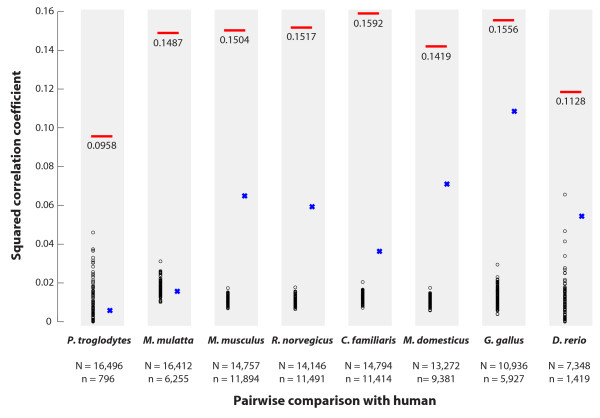
**Genome-wide relationships between log(ω) and log(*ρ*) for eight pairwise comparisons**. Each column reports the details of the genome-wide comparison between human and the indicated species. Below each species, the number of orthologous protein pairs obtained from Ensembl (N) and the number of pairs retained after exclusion (n) are shown. The squared correlation coefficient (R^2^) between log(*ρ*) and log(ω) is shown and plotted in red. The R^2 ^between log(*Ka_I_*/*Ka_A_*) and log(*Ka*) is plotted in blue. The R^2 ^values between log(*ρ*) and log(ω) obtained for each of 100 genome-wide permutations are plotted in black. The observed values are uniformly larger than those obtained via permutation.

To test hypothesis (1), we conducted a genome-wide comparison between each human protein-coding gene and its ortholog, when present and unambiguous, across eight vertebrate species (Figure 3). We restricted ourselves to unique orthologs as designated by Ensembl (see Methods) and used their previously computed alignments. For each alignment, we used the model of Yang and Nielsen [15] as implemented in PAML to compute *ω, ω_I _*and *ω_A _*as described above. Each aligned pair of orthologs thus provides a (*ω, ρ*) coordinate pair that can be entered into a species-specific scatterplot of genes. These eight scatterplots - one for each non-human species in the phylogeny of Figure 3 - show a consistent, non-linear monotonic trend; as *ω *decreases, so too does *ρ*, indicating that the degree to which non-synonymous changes cluster increases with the strength of purifying selection (data not shown). When the two axes are log-transformed, so that log(*ρ*) is plotted against log(*ω*), the relationship becomes linear and highly significant. In Figure 4, log(*ρ*) is plotted against log(*ω*) in blue for 11,894 aligned pairs of orthologous genes identified in human and mouse (see Methods for inclusion criteria). The linear trend depicted in black is highly significant (*r *= 0.3878; p-value < 2.2e-16) and is not limited to the comparison of human and mouse. Indeed, as Figure 5 shows, each of the eight comparisons provides strong evidence of a significant log-linear trend relating our chosen measures of selection and dispersion. To isolate the effect of calibrating by synonymous substitution rates, Figure 5 also includes results from alternative measures of selection and dispersion based on unscaled protein divergence. These results, which compare log(*Ka_I_*/*Ka_A_*) to log(*Ka*) without regard to synonymous substitution rates, show a similar but weaker trend.

To emphasize the significance of our findings, the scatterplot of Figure 4 in red presents a control. Our control, constructed in the spirit of the dispersion ratio, follows the construction illustrated in Figure 2 for synonymous rather than for non-synonymous changes. Thus, whereas *ρ *is created by first partitioning sites in the alignment according to the location of non-synonymous changes, the synonymous dispersion ratio *ρ_S _*is created by first partitioning sites according to where synonymous changes are observed. Figure 4 plots log(*ρ_S_*) against log(*ω*) in red for the human/mouse comparison. As the figure shows, the relationship is not significant (*r *= -0.0156; p-value = 0.087), suggesting that in strong contrast to non-synonymous changes, the clustering of synonymous changes does not depend on the intensity of purifying selection on the protein sequence. As a final validation, we turned to a permutation-based approach whereby the order of sites in each alignment was shuffled. The effect of this, for any one aligned pair of orthologs, is to hold *ω *fixed while varying *ρ *in a random, non-biological way. Permuting each aligned human/mouse pair creates an alternative version of the blue scatterplot in Figure 4; the observed correlation can be thought of as a sample from a null distribution under which selection and dispersion are not biologically related. We used 100 genome-wide permutations to perform a non-parametric test of the null hypothesis that the observed correlation between *ω *and *ρ *is consistent with a spatially random placement of non-synonymous changes. Owing to edge effects and the discrete nature of the data, the expected correlation of *ω *and *ρ *under the null hypothesis is biased away from zero; nevertheless, the correlation observed in our original data is uniformly and substantially larger than any of the permuted realizations (i.e. p-value < 0.01, see Figure 5), and this persists regardless of the comparison. This, once again, supports the existence of a genome-wide trend relating the intensity with which purifying selection acts on a protein and the intensity with which non-synonymous changes are clustered.

### Genes under recent positive selection deviate from the trend

In a pairwise comparison of protein-coding sequences, it is difficult to disentangle the mode and tempo of the evolutionary process. For example, genes under recent positive selection in the human lineage may not appear as such in a pairwise comparison if purifying selection is acting upon the gene in the sister lineage. Put another way, the pairwise comparison reflects the aggregated effects of two evolutionary regimes, one in which the protein evolves at a rate faster than expected under neutrality, and one in which the protein evolves at a rate slower than expected under neutrality. As a consequence of this aggregation, the individual regimes that compose such a mixed regime may be obscured, unless of course additional information is incorporated in the analysis. We hypothesize that the dispersion ratio provides useful information toward disentangling mixed evolutionary regimes. Evidence of this comes from the observation that both purifying selection and positive selection appear to promote the clustering of non-synonymous changes: if both regimes promote clustering, than the degree of clustering observed under a mixed regime may be surprisingly large given the apparent intensity of selection. The stable relationship between log(*ρ*) and log(*ω*) presented previously suggests that log(*ω*) can be predicted from log(*ρ*); in a pairwise comparison that spans a mixed regime, log(*ω*) may be appear too large when compared to a prediction based on the value of log(*ρ*) that was observed. In other words, we hypothesized that a mixed regime might lead to evolutionary rates that are "too fast" for the degree of clustering observed.

**Figure 6:**
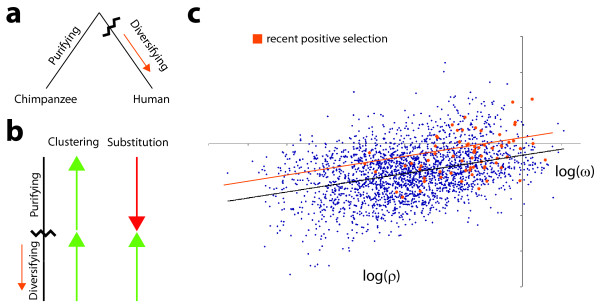
**Deviation of genes under recent positive selection in humans**. **(a) **Illustrated mode of evolution for a gene shared by human and chimpanzee that is under constant positive selection in the human lineage. **(b) **Putative effect of evolutionary mode on intensity of clustering and intensity of non-synonymous change. The rate of substitution is different under diversifying and purifying selection, however both may promote the clustering of changes along the sequence. The arrows convey the intuition: if both modes of selection promote clustering (green upward) while purifying selection yields comparatively fewer substitutions (red downward), then genes under diversifying positive selection should be evolving more rapidly than their degree of clustering predicts based on the overall genomic trend. **(c) **Plot of log(ω) vs. log(*ρ*) for human/chimpanzee orthologs. Genes annotated as being under selection in the human lineage are highlighted in orange. In black is the fitted line $y=\hat{\alpha }+\hat{\beta }x$; in orange is the fitted line $y=\hat{\alpha }+\hat{\gamma }+\hat{\beta }x$. Both *γ *and *β *were found to be significantly larger than zero.

As a test of this hypothesis, we turned to a set of protein-coding genes implicated as being under positive selection in the human lineage after the human/chimpanzee split [18,19]. Reversing the axes from Figure 4, in Figure 6 we identified these genes in a human/chimpanzee scatterplot of log(*ω*) vs. log(*ρ*) (see Methods). Qualitatively, the positively-selected genes (in orange) appear to have larger-than-average values of *ω *for any given *ρ*; quantitatively, we assessed this using a linear model that includes an indicator variable. Letting *X_i _*and *Y_i _*denote the log(*ρ*) and log(ω) values for gene *i*, respectively, and defining the indicator to be *P_i _*be equal to one if gene *i *was deemed to be under recent positive selection and equal to zero otherwise, we tested whether *γ *= 0 in the linear model *Y_i _*= *α *+ *βX_i _*+ *γP_i _*+ *ε_i_*. We were able to reject the null hypothesis *γ *= 0 when tested against the biological one-sided alternative *γ *< 0 (p-value < 0.00467), concluding that as compared to the overall clustering trend the rates of "mixed-regime" genes appear to be elevated.

### The dispersion ratio is a non-redundant predictor of evolutionary rate

Recall that, as depicted in Figure 2a, one interpretation of the dispersion ratio is that it captures the latent segmentation of rate classes within a protein sequence. This segmentation, in turn, may be due to constraints on a protein's structure and function. Viewed in this way, it is not unreasonable to consider the dispersion ratio as a crude but informative surrogate of the structural constraints acting upon a protein. We have provided evidence that this structural surrogate is predictive of the rate at which a protein evolves (i.e. *ω*), and we have shown that the clustering measured by *ρ *is independent of ω when the sequences have been permuted (i.e. in the absence of structuring). In this section, we investigate how *ρ *compares with other established correlates of evolutionary rate.

We have structured this comparison to bring it in accord with the literature. The manuscripts we sought to parallel collectively introduce a diverse set of potential correlates of a protein's evolutionary rate. The measures we consider span a wide range of protein-related attributes, including mRNA expression level [20], protein abundance [21], translational efficiency (as measured by the codon adaptation index) [22,23], dispensability (i.e. fitness when deleted) [24,25], sequence length [26], the number of protein-protein interaction partners [27], the protein's contact density [28], the fraction of residues in the protein that are at least 25% buried, and the fraction of residues involved in various secondary structure elements (helix, strand, turn, coil) [28]. In addition to correlating these attributes both to log(*ρ*) and log(*ω*), we considered each as a controlling variable to test the persistence of a significant log-linear relationship between *ρ *and *ω *in yeast.

The yeast dataset we employ comes from [16] and includes annotated protein-coding genes from four *Saccharomyces *species: *S. cerevisiae, S. paradoxus, S. mikitae*, and *S. bayanus*. We again focused on groups of unique orthologs, and because here for each protein-coding gene we have four sequences instead of two, we were forced to extend the dispersion ratio beyond pairwise comparisons. Our approach was to treat the unrooted phylogeny from [16], ((*S. cerevisiae, S. paradoxus*), (*S. mikitae, S. bayanus*)), as representing five separate pairwise comparisons to be aggregated (though see Discussion for alternatives). To accomplish this required us to infer the sequences at the internal nodes of the tree, and we did so under a probabilistic model from [29], using the algorithm of [30]. For each pair of sequences spanning a branch on the tree, we partitioned their alignment as in Figure 2 to obtain four values: (1) *Ka_A_*, the adjacent rate of non-synonymous changes, (2) *Ks_A_*, the adjacent rate of synonymous changes, (3) *Ka_I_*, the isolated rate of non-synonymous changes, and (4) *Ks_I_*, the isolated rate of synonymous changes. Note that whereas before we combined these to compute *ρ*, here we have kept them separate so that each can be summed across the tree. In this way, we computed the dispersion ratio for each yeast protein-coding gene as (Σ*Ka_I_*/Σ*Ks_I_*)/(Σ*Ka_A_*/Σ*Ka_A_*).

As before, we find a highly significant log-linear relationship between the dispersion ratio and evolutionary rate. To test whether or not that relationship persists after controlling for the aforementioned protein-related attributes, we used the method of partial correlation. Specifically, we computed the partial correlation between log(*ρ*) and log(*ω*) after controlling for each of the protein-related attributes in Table 1. The results show that the log-linear relationship between selection and dispersion remains highly significant even after controlling for a variety of established evolutionary correlates. The strength of that relationship, in comparison to those observed for other attributes, is remarkable (see Table 1) and suggests that the dispersion ratio is capturing an important determinant of evolutionary rate.

## Discussion

As a protein-coding gene evolves, non-synonymous substitutions do not accumulate uniformly along its sequence. There is heterogeneity among the rates at which individual sites within a protein evolve, and part of that heterogeneity is induced by structural and functional constraints. Though the structural and functional domains that comprise proteins are contingent upon tertiary folding, there is enrichment within domains for residues that are contiguous along the primary sequence. As such, within proteins there exists rate autocorrelation that can be, and has been, exploited to annotate regions of putative importance.

In a pairwise comparison of protein-coding genes, rate heterogeneity manifests in the non-random placement of non-synonymous changes. One expects a dearth of such changes in regions of structural and functional importance and a relative excess where the intensity of selection is less. The aggregation of changes outside of important regions may lead to the appearance that non-synonymous changes are clustering. We speculated that the appearance of clustering would increase with an increasing intensity of selection, and we developed the dispersion ratio to test that hypothesis. Confirming our speculation, we found a highly-significant log-linear relationship between the dispersion ratio and evolutionary rate. This relationship was observed to be robust to both choice of species and degree of evolutionary divergence.

Just as purifying selection acts to cluster substitutions along the sequence of a protein, there is evidence that diversifying selection leads to clustering as well. This led us to consider the case of genes whose modes of evolution differ on sister lineages. In cases when the evolutionary trajectory spanned by a pairwise comparison contains a mixture of purifying and diversifying selection, we hypothesized an effect on the relationship between the dispersion ratio and evolutionary rate. Having already observed that the degree to which non-synonymous changes cluster is predictive of the rate at which a protein is evolving, we reasoned that for mixed regimes such predictions would be biased downward. At least for the data we examined, this turned out to be the case: for genes under positive selection in the human lineage, the evolutionary rate estimated from a human/chimpanzee comparison was greater than what the degree of clustering would predict.

To place in perspective the contribution of the dispersion ratio as a predictor of evolutionary rate, we compared its explanatory power to those of a diverse set of protein-related attributes. In doing so, we found log(*ρ*) to be a highly-significant and non-redundant correlate of the logarithmic rate, log(*ω*). The correlation between log(*ρ*) and log(*ω*), and its persistence after conditioning on other correlates of evolutionary rate, speaks to either a determinant of evolutionary rate that has not yet been characterized or a deficiency in the way evolutionary rate has been quantified in this particular set of studies. Whatever the case, it appears that non-synonymous clustering is a reliable, non-redundant, sequence-based predictor of *ω*.

Because the dispersion ratio behaves differently under neutrality and under purifying selection, and because permutations can be used to populate a sensible null distribution, one can envision using the dispersion ratio in a test of selection. Nevertheless, we did not devise *ρ *as a statistic to test the behavior of individual genes, and such tests, though conceivable, would likely be underpowered and inferior to existing methods (e.g. [12,31]). These methods, unlike ours, were specifically designed to identify the presence of clustered substitutions and test their significance against an appropriate null hypothesis about a specific gene. By contrast, we were motivated by simplicity and proposed the dispersion ratio as an intuitive means of testing the existence of genome-wide evolutionary trends, without regard to any particular gene. Other measures of clustering are likely to perform similarly, and indeed we observe similar results to those presented when *ρ *is replaced by a model-based measure of autocorrelation (taken from [32]; data not shown).

The intuition behind our statistic and its relationship to evolutionary rate is grounded in dependencies induced by protein tertiary structure. Though *ρ *is a function of sequence and not structure, the dispersion ratio, like the methods from which it was inspired (e.g. ET, ESF, SWAKK), leverages the fact that adjacent residues in the sequence are structurally proximal. It seems reasonable that a structurally-informed analog of the dispersion ratio would be superior to *ρ *in validating the hypotheses of this manuscript, but we did not find this to be the case (data not shown). This may be due to, among other possibilities, the limited number of structures available or the manner in which we extended our statistic.

In interpreting the results presented here, it should be noted that all of our analyses were contingent upon sequence alignment. Because alignment uncertainty tends to increase with sequence divergence, to the extent that alignment errors affect neighboring sites, one expects a spurious non-biological correlation between *ω *and *ρ*. While alignment error may indeed contribute to the signal we observe, we do not believe it to play a major role. Several of our analyses feature very closely related species whose orthologous proteins are predominantly the same length. For these proteins, the alignment is unambiguous, unless there was both an insertion and deletion event.

## Conclusions

In summary, we have proposed a simple statistic that quantifies the degree of non-synonymous clustering in a pairwise comparison, and we did so to test hypotheses about how clustering varies with evolutionary rate. We found ample evidence of a strong log-linear relationship, and we tested the robustness and validity of our observations in a number of ways. To investigate generality, we considered eight vertebrate pairwise comparisons spanning a wide range of evolutionary divergence, as well as a comparison of four *Saccharomyces *yeast. To investigate potential artifacts, we used as controls both a permutation approach and a synonymous dispersion statistic. To investigate methodological dependence, we considered alternatives to the dispersion ratio, including the idea of simply "counting" synonymous and non-synonymous changes as suggested by Nei and Gojobori [33] and by Li [34] (data not shown). In every case, for every comparison, we find that non-synonymous clustering intensifies with increasing purifying selection. The ubiquity of this relationship supports the concept of a loose segmentation model for protein sequences as well as the use of *de novo *annotation methods that have implicitly capitalized upon it.

## Authors' contributions

LGM and EAS conceived the study, performed the statistical analysis, participated in the design and drafted the manuscript. All authors read and approved the final manuscript.
